# Genomic Comparative Study of Bovine Mastitis *Escherichia coli*

**DOI:** 10.1371/journal.pone.0147954

**Published:** 2016-01-25

**Authors:** Florent Kempf, Cindy Slugocki, Shlomo E. Blum, Gabriel Leitner, Pierre Germon

**Affiliations:** 1 INRA, UMR 1282 Infectiologie et Santé Publique, CIRM-Bactéries Pathogènes, F-37380, Nouzilly, France; 2 INRA, UMR 1282 Infectiologie et Santé Publique, Immunité et Infections Mammaires, F-37380, Nouzilly, France; 3 Université François Rabelais de Tours, UMR 1282 Infectiologie et Santé Publique, F-37000, Tours, France; 4 National Mastitis Center, Division of Bacteriology, Kimron Veterinary Institute, POB 12, Bet Dagan, 50250, Israel; Centre National de la Recherche Scientifique, Aix-Marseille Université, FRANCE

## Abstract

*Escherichia coli*, one of the main causative agents of bovine mastitis, is responsible for significant losses on dairy farms. In order to better understand the pathogenicity of *E*. *coli* mastitis, an accurate characterization of *E*. *coli* strains isolated from mastitis cases is required. By using phylogenetic analyses and whole genome comparison of 5 currently available mastitis *E*. *coli* genome sequences, we searched for genotypic traits specific for mastitis isolates. Our data confirm that there is a bias in the distribution of mastitis isolates in the different phylogenetic groups of the *E*. *coli* species, with the majority of strains belonging to phylogenetic groups A and B1. An interesting feature is that clustering of strains based on their accessory genome is very similar to that obtained using the core genome. This finding illustrates the fact that phenotypic properties of strains from different phylogroups are likely to be different. As a consequence, it is possible that different strategies could be used by mastitis isolates of different phylogroups to trigger mastitis. Our results indicate that mastitis *E*. *coli* isolates analyzed in this study carry very few of the virulence genes described in other pathogenic *E*. *coli* strains. A more detailed analysis of the presence/absence of genes involved in LPS synthesis, iron acquisition and type 6 secretion systems did not uncover specific properties of mastitis isolates. Altogether, these results indicate that mastitis *E*. *coli* isolates are rather characterized by a lack of bona fide currently described virulence genes.

## Introduction

*Escherichia coli* is one the main pathogens involved in cases of bovine mastitis. This common dairy disease is of major economic interest [1–3]. Mastitis caused by *E*. *coli* is of acute onset, yet the time for recovery of the gland may be long, during which milk composition remains affected thus extending the economic impact of this pathogen in dairy production [4]. The *E*. *coli* species is very diverse comprising commensal strains as well as pathogenic strains clustered in different pathovars based on clinical data and and specific virulence properties. Strains belonging to intestinal pathogenic pathovars (IPEC) are able to colonize the host intestine and can cause a large variety of symptoms ranging from mild diarrhea to severe dysentery [5]. On the other hand, extraintestinal pathogenic *E*. *coli* (ExPEC) strains are involved in several diseases including urinary tract infections and neonatal meningitis [6–8]. A Mammary Pathogenic *E*. *coli* (MPEC) pathovar has been suggested to include strains associated with mastitis in dairy animals, but still needs to be validated by the identification of MPEC specific traits [9].

The genetic bases underlying the classification of strains in a specific pathovar are partly known. For example, specific virulence factors including toxins, autotransporters, type II and III effectors were found in association with distinct pathovars [5]. But a more complex picture progressively emerged. Close relationships may be observed among strains belonging to distinct pathovars, as a result of genomic plasticity and horizontal acquisitions [8, 10]. Other studies have questioned the specificity of virulence factors. This is the case for the Stx-toxin, which was considered as a typical virulence factor of EHEC (enterohemorrhagic) strains, but was also detected in the EAEC (enteroaggregative) *E*. *coli* responsible for the German outbreak of hemolytic-uremic syndrome in 2011 [11].

The definition of an MPEC pathovar, along with criteria specific for these strains, is at the cornerstone of studies focusing on bovine mastitis *E*. *coli* strains. So far, multiple studies have failed to unravel a specific virulence gene set associated with mastitis strains [12–16]. This subsequently underlines the role played by ‘cow factors’ in pathogenicity. A large number of strains may be causative of bovine mastitis [17] and much remains to be done to assess whether they share particular genomic traits.

Concerning MPEC-specific genes, candidates could be associated with the phenotypes relevant for mastitis causative agents: ability to multiply and survive in the mammary gland milieu [15], resistance to phagocytosis and killing by neutrophils [18–20], capacity to stimulate a pro-inflammatory response by mammary epithelial cells through expression of Microbe-Associated Molecular Patterns (MAMPs) [21, 22] or attachment and invasion of mammary epithelial cells [23].

Comparative genomics may allow a better understanding of the genetic basis of these mechanisms. The present paper is an analysis based on the comparison of five genomes of *E*. *coli* strains isolated from cases of bovine mastitis. The aim of this study was to identify genomic properties supporting the MPEC pathovar hypothesis, first by investigating their gene repertoire, second by inferring their phylogenetic relationships to strains of other origins.

## Materials and Methods

### *E*. *coli* strains

A set of 40 *E*. *coli* strains isolated from cases of clinical mastitis was used in preliminary MLST analyses. These strains were isolated in France from cases of severe, peracute mastitis, that is from cows showing local signs of inflammation and systemic signs of infection (fever above 39.6°C and/or loss of apetite and/or prostration). *E*. *coli* strains included in the genomic comparative analyses are listed in Table 1 and included 5 mastitis related strains, isolated either from clinical cases or from a case of persistent mastitis. Strains VL2874, D6-113.29 and D6-117.07 were isolated from cases of severe, per-acute mastitis, whereas strain VL2732 was isolated from a case of persistent mastitis, confirmed by multiple isolation of genotypically similar bacteria from the same quarter over a six month period [13]. Strain P4 was isolated from clinical mastitis in the UK [24] and has become largely accepted as a prototypical mastitis strain for *E*. *coli* mastitis research. Strain K71 was isolated from cow shed [15] and does not cause inflammation in the mammary gland in mice [14] or cows (S. Blum, personnal communication). This strain was included in the analysis along with 13 *E*. *coli* strains from other pathovars chosen to represent the different phylogroups described in the *E*. *coli* species (Table 1). The *Escherichia fergusonii* strain ATCC 35469 was chosen as an outlier in phylogenetic analyses. Genomic DNA sequences were downloaded from GenBank or described in previous reports [14, 25]. Strains P4 (CIRMBP-993), B41 (CIRMBP-546), D6-113.11 (CIRMBP-549) and D6-117.07 (2IM-260) can be obtained upon request at the International Centre for Microbial Ressources—Bacterial Pathogens (CIRM-BP http://www6.inra.fr/cirm_eng/Pathogenic-Bacteria).

**Table 1:** List of strains used in this study.

Strains	Pathotype	Phylogroup ^(a)^	Sequence reference	Reference
*E*. *coli* P4	MPEC (clinical mastitis)	A (ST10)	AJQW00000000.1	[26]
*E*. *coli* VL2732	MPEC (persistent mastitis)	A (ST10)	JTFD00000000	[14]
*E*. *coli* VL2874	MPEC (clinical mastitis)	A (ST10)	JTFE00000000	[14]
*E*. *coli* D6-113.11 ^(b)^	MPEC (clinical mastitis)	E (ST1301)	CCCO000000000.1	[25]
*E*. *coli* D6-117.07 ^(b)^	MPEC (clinical mastitis)	A (ST10)	CCCP000000000.1	[25]
*E*. *coli* K71	cow shed	B1 (ST58)	JTFF00000000	[14]
*E*. *coli* K-12 MG1655	Commensal	A (ST10)	NC_000913.3	[27]
*E*. *coli* ATCC8739	Commensal	A (ST3012)	CP000946.1	(direct submission)
*E*. *coli* B41	ETEC	A	NZ_AFAH02000000	(direct submission)
*E*. *coli* CFT073	ExPEC	B2 (ST73)	AE014075.1	[28]
*E*. *coli* ED1A	Commensal	B2	CU928162.2	[29]
*E*. *coli* G58-1	Commensal	A	NZ_AFDX00000000.1	(direct submission)
*E*. *coli* HS	Commensal	A	CP000802.1	[30]
*E*. *coli* IAI1	Commensal	B1 (ST1128)	CU928160.2	[29]
*E*. *coli* IAI39	ExPEC	F	CU928164.2	[29]
*E*. *coli* S88	ExPEC	B2	CU928161.2	[29]
*E*. *coli* Sakai	EHEC	E	NC_002695.1	[31]
*E*. *coli* SE11	Commensal	B1	NC_011415.1	[32]
*E*. *coli* UMN026	ExPEC	D	NC_011751.1	[29]
*E*. *fergusonii* ATCC 35469			NC_011740	[29]

^(a)^ sequence types (ST) were retrieved from EnteroBase (http://enterobase.warwick.ac.uk)

^(b)^ also known as CIRMBP-549 (D6-113.11) and 2IM-260 (D6-117.07)

### General genomic features

Genome annotation was performed by AGMIAL, an integrated bacterial genome annotation system [33]. Prediction of coding sequences used SHOW, the self-training gene detection software based on hidden Markov models [33].

PlasmidFinder [34] was used to detect plasmid-associated nucleotide sequences. Plasmid-associated genes were next detected using a basic local TBLASTN search (identity cut-off 80%; HSP score coverage cut-off 80%) [35]. For this, a database containing all previously published *E*. *coli* plasmid sequences (retrieved from the ENA database) was used to query the gene nucleotide sequence dataset of each strain. A similar approach was used to detect phage-associated genes using the phage sequences found in the ENA public database at the date of analysis.

### Comparative genomics

Orthologues genes were clustered using OrthoMCL2 [36]. Under this scheme BLASTP alignments were performed within and between all genome pairs (e- value cut-off = 1E-5). The resulting normalized similarity matrix was explored using a Markov Cluster algorithm in order to define clusters containing orthologs and recent paralogs. Briefly, random walks were performed by successive matrix transformations (i.e. expansion and inflations) until reaching an equilibrium state. The resulting doubly idempotent matrix may be next interpreted to delineate distinct clusters [37].

### Comparison to *E*. *coli* pangenome

We retrieved all the *E*. *coli* genes recorded in the Genoscope genomic databases (data kindly provided by David Roche, Genoscope). TBLASTN was used to build the list and detect homologous genes within the *E*. *coli* genomic data available at the date of the comparison (identity cut-off 80%; HSP score coverage cut-off 80%).

The resulting pangenomic dataset was used to query the genomes of the 5 mastitis-associated *E*. *coli* and the 14 strains retained for comparison (Table 1) by TBLASTN (identity cut-off 80%, HSP score coverage cut-off 80%). This yielded a list of genes shared by all of the *E*. *coli* 19 strains whose sequences were concatenated for further phylogenetic analyses. The data relative to the genes only found in a subset of the 19 strains were treated as presence/absence binary bits.

### MLST analyses

The strains under analysis were first compared on the basis of Wirth et al.’s MLST scheme [38]. For the sake of comparison we retrieved the allelic profiles of the 83 ECOR strains from the MLST database managed by the University of Warwick (http://mlst.warwick.ac.uk/mlst/dbs/Ecoli/documents/primersColi_html). The sequences at each locus were extracted from the 5 mastitis strains genomic using BLASTN. An additional set of 40 mastitis-associated strains were characterized by direct sequencing of the seven MLST loci as described elsewhere [38]. These strains are available upon request to the International Centre for Microbial Resources (CIRM-BP; http://www6.inra.fr/cirm_eng/Pathogenic-Bacteria). *Salmonella enterica subsp*. *enterica* ser. Typhi was used as an outlier to root the tree (data taken from [38]).

Sequences were concatenated and aligned using the Run-mummer3 software included in the MUMmer3.0 program suite [39]. Genetic relationships were inferred using a GTR model with the CAT approximation of rate heterogeneity option implemented in RAxML v8.1 (Stamanakis 200). Node support was assessed using the rapid bootstrap procedure (100 bootstraps). Tree visualization was done using Dendroscope 3.2.10 [40].

The expected distribution of the mastitis strains among the 5 phylogroups defined by Selander et al. [41] was estimated using the frequency of each phylogroup within ECOR collection (45[35%], 40[31%], 10[8%], 19[14%] for A, B1, B2, D and E group respectively). The observed distribution was compared using a parametric Chi^2^ test.

### Core genome phylogenetic analyses

The comparison of the pangenomic dataset (see above) to the genomes in the analysis yielded a subset of 1976 genes common to all 19 strains. Homologous gene sequences were retrieved from the *Escherichia fergusoniii* genome dataset using a similar BLAST comparison and used as outgroups in further phylogenetic analyses.

Each of the 1976 genes was first treated separately. The best evolutionary model was inferred for each of them under the GTR+gamma+I hypothesis using PhyML 20111216 [42]. Second, a global composite model was next computed using the mean value of the relevant parameters weighted by gene sizes. Concatenated data were analyzed using this composite model and a quartet puzzling procedure implemented in TreePuzzle 5.2 to estimate branch support [43]. A consensus tree maximizing the branch support was finally constructed using the 50% majority rule.

### Accessory genome

A total of 10862 genes were found in at least one but not all the 19 compared strains. The resulting presence/absence data were summarized by hierarchical clustering using binary distances. The computations were performed using R 2.15.1 [44].

### *E*. *coli* virulence factors

A basic local TBLASTN search for putative virulence genes was performed using a list of 302 virulence genes [13, 45] (S1 Table). The results of the presence/absence matrix obtained were treated using Cluster 3.0 with complete linkage analysis and euclidean distances [46].

### Incongruences between species and individual gene trees

The concatenated sequences of the 1976 common genes were analyzed in order to detect localized incongruence of phylogenetic signature. The topology found using the whole concatenated dataset was used as reference. The likelihood of this particular topology was estimated using TreePuzzle 5.2 under HKY model for any 5kb sliding window (spaced by 250bp) on the concatenated dataset. The likelihoods and the numbers of polymorphic sites were used respectively as dependent and explicative variables in a regression analysis performed on R v.2.15.1. The deviation between observed and estimated likelihood was summarized in a score that took negative values for an observed likelihood lower than estimated. The sizes of incongruent regions were estimated relative to the positions of flanking genes on the *E*. *coli* EDL933 genome sequence.

## Results

### MLSA-based phylogeny

In order to gain a broad view of the phylogenetic relationships between mastitis *E*. *coli* strains and *E*. *coli* strains of other pathovars, we determined the MLST allelic profile of a collection of 40 strains isolated from cases of clinical mastitis, in addition to 5 mastitis strains for which the complete genome was sequenced and analyzed as discussed below. In order to gain a better resolution of the genetic relationships between strains, the DNA sequences of alleles of these strains were compared to that of strains from the ECOR collection [47].

**Fig 1:**
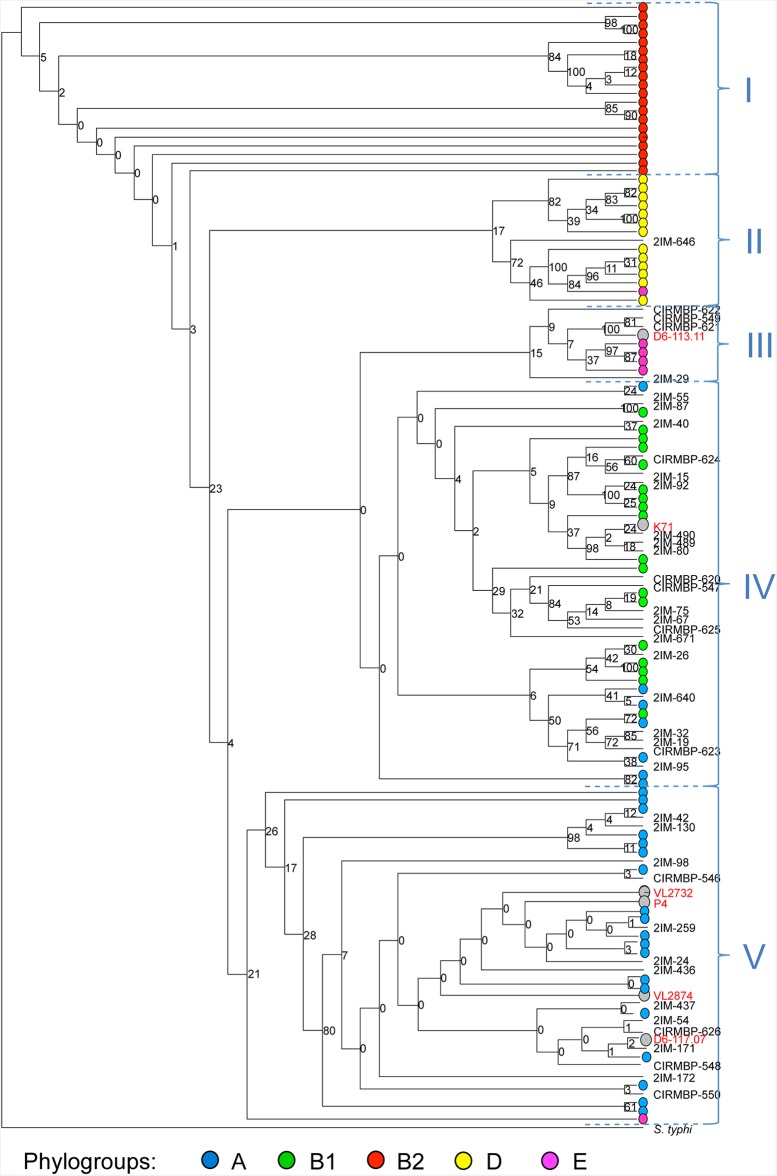
MLST analysis of phylogenetic relationships between mastitis and ECOR *E*. *coli* strains. The 5 fully sequenced mastitis-related strains and strain K71 (grey dots; names in red) and 40 additional mastitis strains were characterized by MLST. *E*. *fergusonii* was used as outgroup. Strains from the ECOR collection are represented by dots colored depending on the phylogroups historically defined within the ECOR collection (Herzer 1990). A, B1, E and D strains were mostly clustered within four monophyletic groups (respectively V, IV, III and II) whereas B2 strains were found in a basal in a basal paraphyletic group (I). The branch length separating the outgroup *S*. *enterica* Typhi is not to scale.

The inference of phylogenetic relationships yielded consistent observations with the historical study of Selander et al. [41] and Herzer et al. [48] (Fig 1). The strains belonging to phylogenetic groups A, B1, B2, D and E were mostly found clustered within four monophyletic and a paraphyletic clade (respectively V, IV, I, III and II; Fig 1). As in previous phylogenetic studies [49, 50], the group B2 seems to have emerged first, followed by D, E and sister groups A and B1. The relationships among the closely related strains belonging to group A, the more recently evolving branch, are not well resolved and show only weak support. Mastitis-associated strains were found across 4 of the 5 phylogroups. A total of 18 (41%), 21 (48%), 5 (9%) and 1 (2%) mastitis strain fall within clades predominantly including A, B1, E and D strains. As previously analyzed, K71 strain clustered with B1 strains [14]. The general distribution of the mastitis strains is biased toward a higher frequency in A and B1 phylogroups (X^2^ test *p*-value = 0.01).

### Genome repertoire comparison

Five recently sequenced strains of bovine mastitis-related *E*. *coli* (D6-113.11, D117.07 [25], P4 [26], VL2732 and VL2874 [14]) and one strain non-pathogenic in the mammary gland (K71 [17]) were used for the present study. The fully sequenced strains VL2732, VL2874 and D6-117.07 were strongly related to the mastitis model strain P4 and belong to the phylogroup A (Fig 1). The closest relative of D6-113.11 and K71 were respectively E and B1 strains. The general features are summarized in Table 2. The genome length was comprised between 4.66 and 5.11 Mb; the number of annotated genes ranged between 4522 and 4982, including putative plasmid genes. The G+C contents were very close to the value usually found in *E*. *coli* strains (50.8% for MG1655). A number rRNA and tRNA genes of the same order of magnitude was detected (respectively 4–8 and 68–73, against 22 and 89 in MG1655). Estimated numbers of genes located on plasmids, of insertion sequences (IS), phage- or plasmid- associated genes did not reveal strong differences between the newly sequenced mastitis strains and other *E*. *coli* (Table 3) [29].

**Table 2:** General characteristics of whole-sequence datasets of *E*. *coli* strains of the present study.

Strain	*GC%*	*(total size in bp)*	rRNA genes	tRNA genes	Putative proteins	Assigned function	Hypothetical proteins	IS-like	Phage associated
D6-117.07	50.78	5097170	5	68	4557	3729	828	88	24
D6-113.11	50.51	4795662	8	59	4804	3760	1071	24	68
P4	50.56	4872465	8	65	4645	3979	666	43	22
VL2732	50.65	4665867	4	75	4522	3720	885	29	15
VL2874	50.54	4786508	5	73	4650	3729	828	88	24
K71	50.67	5117165	4	69	4982	3760	1071	24	68

**Table 3:** Plasmid related genes found in the genome of *E*. *coli* strains of the present study.

Strain	Putative proteins	Assigned function	Hypothetical proteins	IS-like	Phage associated
D6-117.07	126	104	22	73	1
D6-113.11	108	81	27	13	2
P4	120	102	18	31	1
VL2732	100	74	7	20	1
VL2874	126	104	29	32	1
K71	47	41	27	20	1

### Core genome based phylogeny

**Fig 2:**
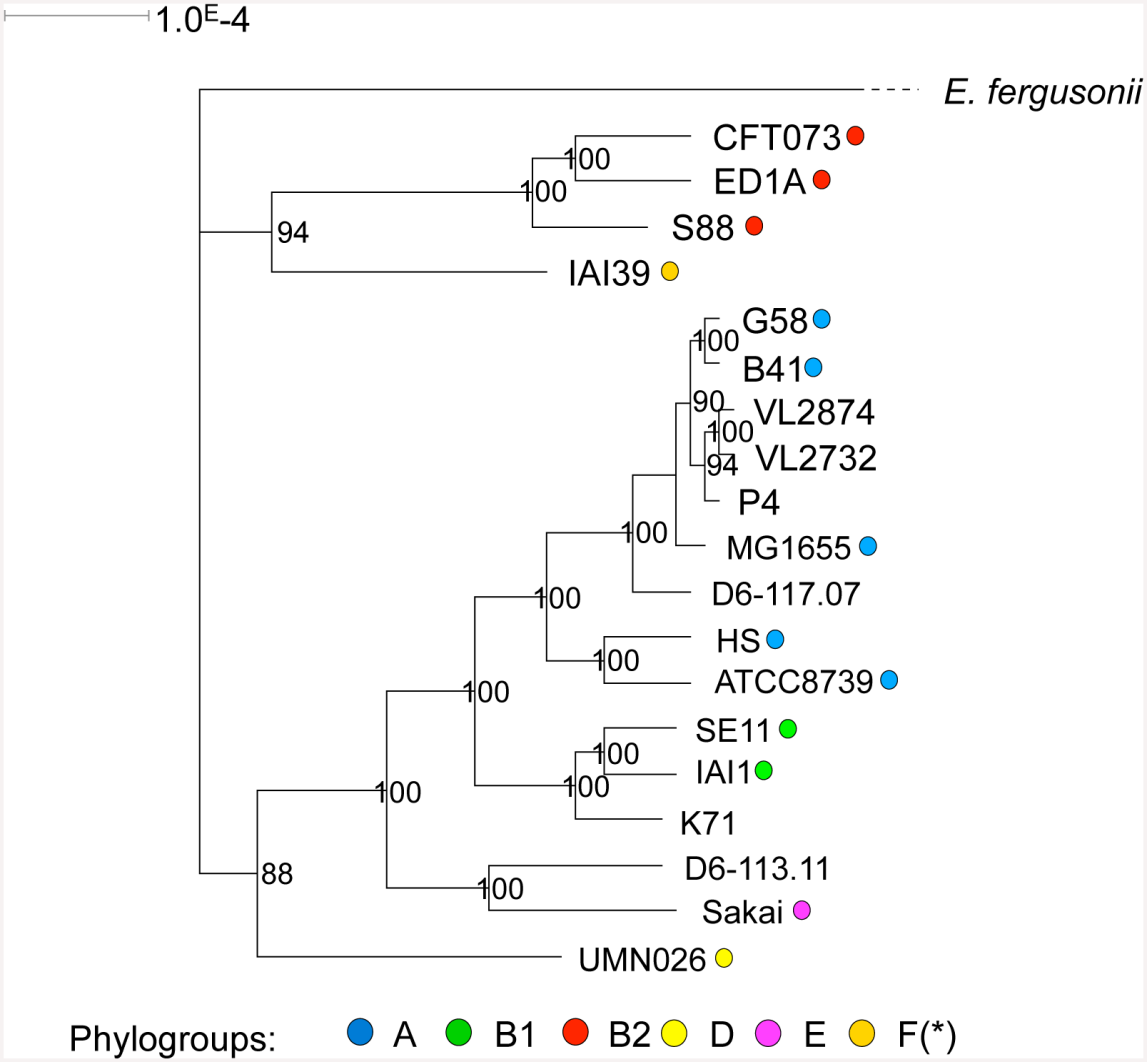
Maximum likelihood phylogenetic analysis. Analysis was performed on the 5 fully sequenced mastitis strains and 14 additional genomes including those of bovine mastitis model strain P4. The concatenated sequence of the 1976 shared genes were compared under a GTR+I model hypothesis. Branch support are estimated by quartet puzzling (1000 steps). Phylogroups are represented with colored dots. The branch length separating *E*. *fergusonii* is not to scale.

The genome repertoire of *E*. *coli* strains can be separated into two sets: a core genome including genes present in all strains and an accessory genome that confers specificity to each *E*. *coli* strain. As a first step, we analyzed the core genome of the mastitis isolates: the pangenomic data for the *E*. *coli* species retrieved from the Genoscope public database was compared to our dataset including the 5 genomes of mastitis strains and 14 published *E*. *coli* genomes. The concatenation of the 1976 genes shared by all the 20 genomes under scrutiny yielded a 1 875 936 bp sequence for each strain. A strongly supported phylogeny was found by comparing these sequences (Fig 2). The inferred phylogenetic tree is in accordance with the MLST-based approach: P4, D6-117.07, VL2732 and VL2874 were clustered with phylogroup A strains within a single monophyletic clade. K71 was more closely related to phylogroup B1 strains and D6-113.11 to the phylogroup E strain Sakai.

### Accessory genome analysis

**Fig 3:**
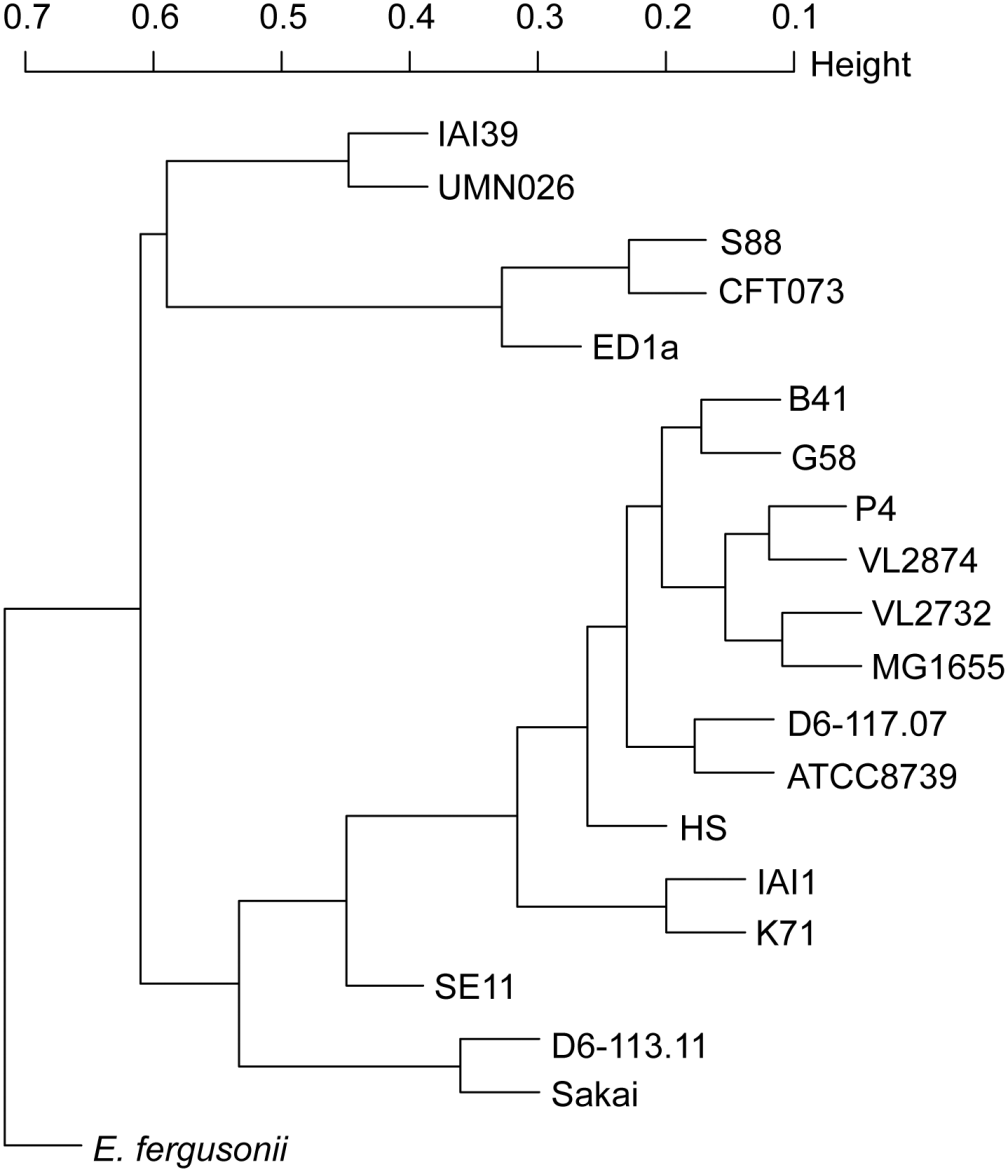
Hierarchical clustering based on the presence of accessory genes. The binary presence/absence was used to compute the distance among each pair of strains.

We then attempted to analyze the relatedness of mastitis isolates in terms of accessory genome. A hierarchical clustering was performed after analysis of the presence/absence of each gene of the pangenomic dataset in each of the strains included in our analysis. One should note that very similar clustering patterns were observed using the common genes phylogenetic approach and accessory genes presence/absence. *E*. *fergusonii*, the B2 strains cluster and the strains representing the F and D phylogenetic group (respectively I1I39 and UMN026) were in basal positions (Fig 3). A larger cluster included the remaining *E*. *coli* with the E (Sakai) and B1 (IAI1 and SE11) strains was located basally. The mastitis *E*. *coli* were clustered within the same phylogenetic group as determined above (A for P4, D6-117.07, VL2732 and VL2874; E for D6-113.11) and strain K71 clustered with group B1. These results do not argue for a large-scale acquisition of mastitis related-specific genes.

We then tried to identify MPEC specific genes by orthologous gene clustering. This analysis yielded a total of 88 clusters that included genes taken from 2 or more of the 5 mastitis strains. For 59 of these clusters we nevertheless found a homologous gene in the pangenomic dataset (see below) which means that they were already found in a previously studied *E*. *coli* strain. These clusters were removed from the analysis. We then kept a total of 29 clusters that included exclusively genes taken from respectively 2 of the 5 mastitis strains. A final BLASTP search was performed by querying public databases in order to detect homologous genes. This step allowed to detect close homology for all except for two of the genes included within the putative mastitis-related clusters (identity percentage > 90%; coverage > 90%) (S2 Table).

As a second approach to identify MPEC-specific genes, we attempted to detect strain specific genes in each of the mastitis strains. This was first done by comparing their genomic data to an *E*. *coli* pangenomic dataset. The TBLASTN search yielded a total of 675, 590, 365, 350 and 595 putative orphan genes (respectively found in D6-113.11, D6-117.07, VL2874, VL2732 and P4). Short sequences (<200 pb) with no assigned function were considered as potential pseudogenes and removed from the analysis.

The pangenomic dataset used for comparison included a single sequence for each gene. This first step was thus likely to produce false orphans genes for highly polymorphic gene loci and was thus refined using the 19 *E*. *coli* genomes for comparison. This second comparison allowed detection of homologous genes for all except for 90, 47, 75, 98 and 92 genes (respectively found in D6-113.11, D6-117.07, VL2874, VL2732 and P4).

A final BLASTP search was performed by querying public protein sequence databases in order to broaden the homologous gene search among all previously published *E*. *coli* genetic data. This allowed to find a close homologous gene (identity percentage > 90%; coverage > 90%) for all except 1, 12, 8, and 15 genes for D6-113.11, VL2874, VL2732 and P4, respectively (S3 Table). These 36 genes are therefore the only genes that could be considered as mastitis isolate specific, although they are not present in every mastitis isolate.

**Fig 4:**
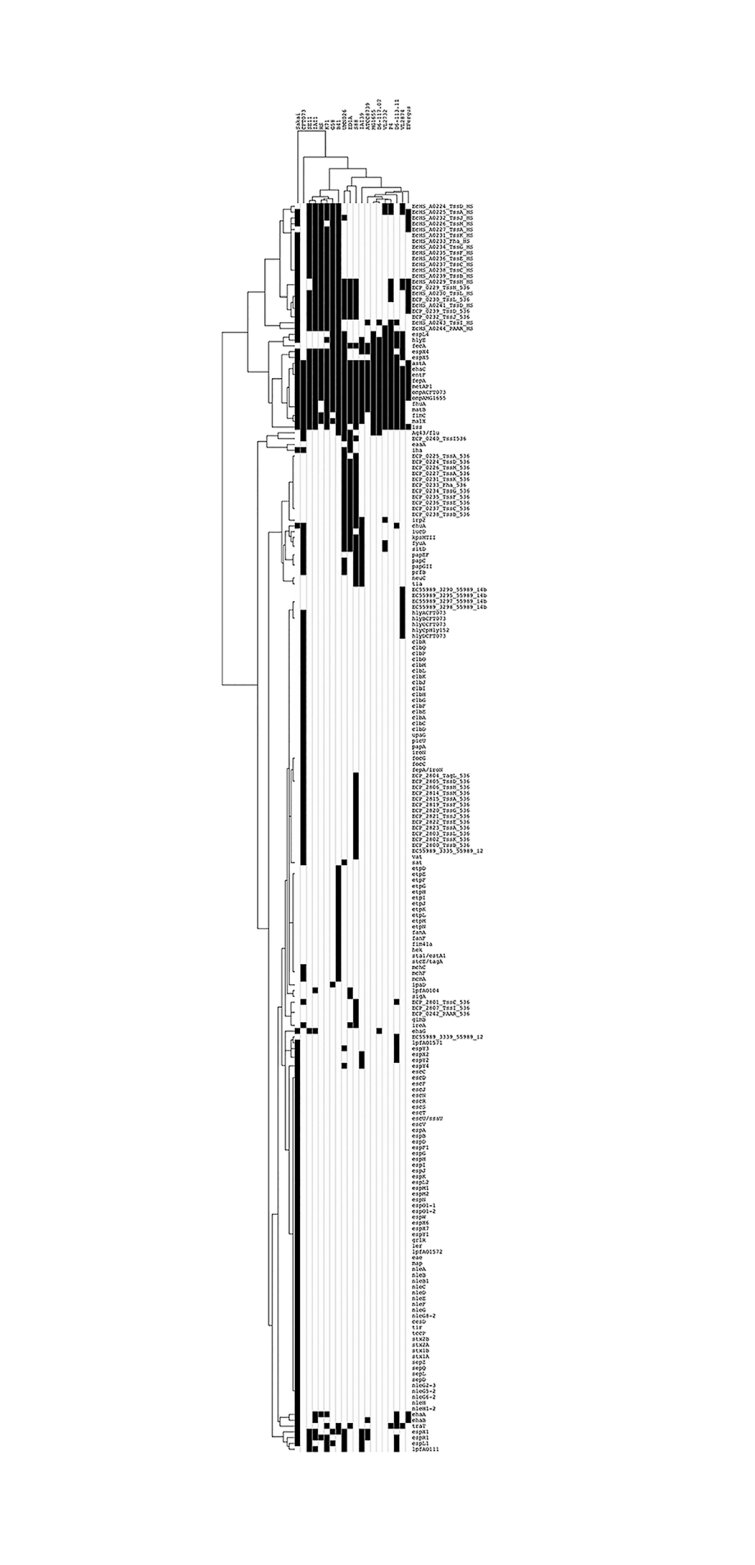
Hierarchical clustering based on the presence of potential virulence genes. Presence/absence matrix of a set of 302 potential virulence genes was analyzed using tblastn (black box indicates presence). Hierarchical clustering of the results was performed using Cluster 3.0 and were visualized using Java Treeview.

Finally, a gene-candidate analysis was performed to assess the presence/absence of 302 candidate virulence genes in the 19 *E*. *coli* strains under comparison. Only a few of these genes were detected in mastitis isolates (Fig 4). Hierarchical clustering indicated that mastitis isolates P4, D6-117.07, D6-113.11, VL2874 and VL2732 cluster with the K-12 MG1655 strain. Although the prevalence of ExPEC specific genes in mastitis isolates is rather low, a few key features of this analysis are worth noting.

First, as noted in previous studies, the *fecA* gene is present in all mastitis isolates, as in MG1655, but not strain K71, a strain isolated from cow shed that was recently described as not able to induce mastitis in mice [14]. Whether this contributes to mastitis remains to be explored. Indeed, other iron acquisition systems such as the enterobactin system are present in mastitis isolates (see below).

Second, a link between long polar fimbriae and mastitis isolates has been suggested [51]. Several long polar fimbriae genes have been identified in the *E*. *coli* species, which were named here *lpfA_O104* (a.k.a. *lpfA141*), *lpfA_O111* (a.k.a. *lpfA154*), *lpfA_O157_1* and *lpfA_O157_2* based on the serogroup of the strain from which the sequences were extracted. None of these genes were found in phylogroup A strains P4, D6-117.07, VL2732 and VL2874. Only *lpfA_O111* and *lpfA_O157_1* were found in strain D6-113.11 while only *lpfA_O111* was found in strain K71.

Third, type 6 secretion systems (T6SS) have been identified in other mastitis isolates [16]. The presence of five different types of T6SS was analyzed using the SecReT6 database [52]. The T6SS subtype i2 from strain 536 was not observed in any of the mastitis isolates. Only one or two genes homologous to T6SS subtype i1 from strain 536 were observed in strains VL2874 and P4, respectively. Concerning the T6SS from strain HS (subtype i1), only a few genes were observed in mastitis isolates, suggesting that these systems might not be complete and fully functional. On the contrary, the T6SS from strain HS was present in the genome of strain K71. Four genes (out of 14) encoding components of the T6SS subtype i4b from strain 55989 were found in strain VL2874. To summarize, these results show a rather low prevalence of T6SS systems in mastitis isolates and therefore question the role these systems could play during mastitis.

**Fig 5:**
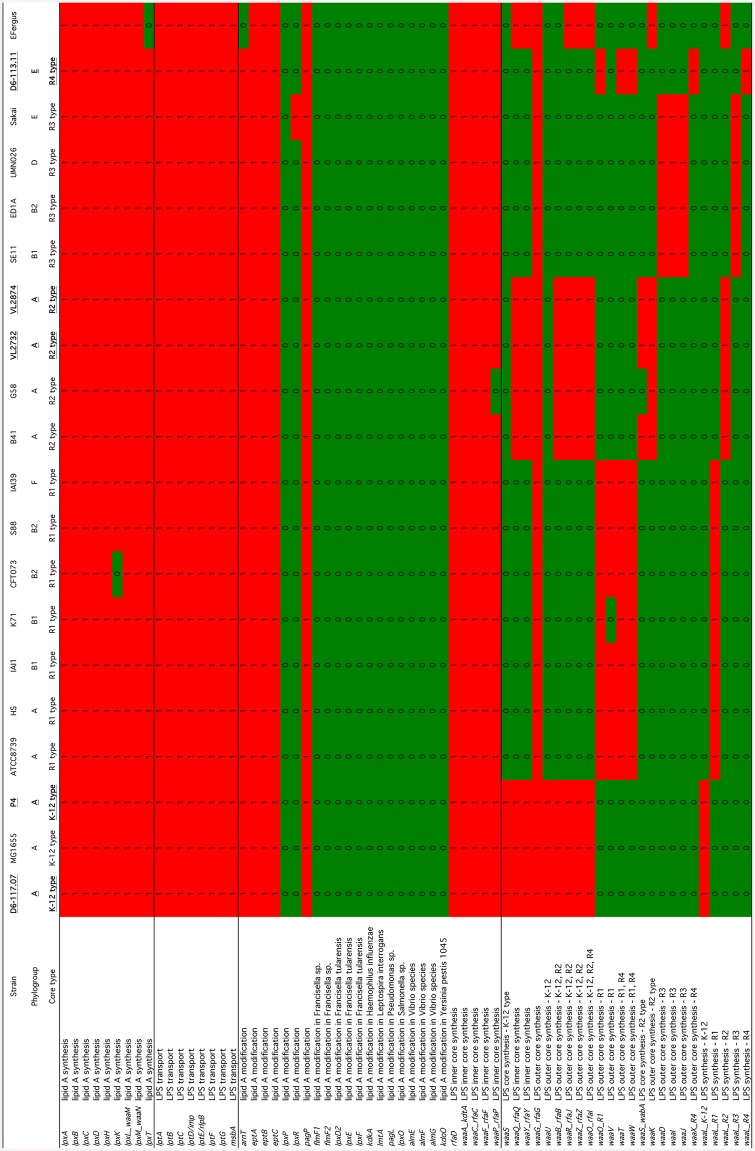
Presence/absence matrix of genes involved in LPS synthesis. Presence was analyzed using tblastn (red indicates presence, green indicates absence). Mastitis isolates are in bold characters and underscored.

Given the importance of LPS in the response of cows to an *E*. *coli* infection, we analyzed in more details the presence/absence of the genes involved in LPS synthesis (Fig 5). LPS is composed of three different regions, namely, from inside to outside, the lipid A, the oligosaccharide core region and the O-antigen [53–55]. As can be seen on Fig 5, lipid A synthesis genes are all present as well as a conserved set of genes involved in lipid A modification. Based on these data and according to published data, the type of core oligosaccharide can be inferred [56]. Mastitis isolates P4 and D6-117.07 are predited to possess a K-12 core, VL2874 and VL2732 an R2 type core, strain D6-113.11 an R4 type core and strain K71 an R1 type core. We cannot therefore assign mastitis isolates to one specific type of core oligosaccharide. Our data also do not argue for the presence of specific lipid A modifications that would interfere with the recognition of *E*. *coli* by the immune system of the host [57].

**Fig 6:**
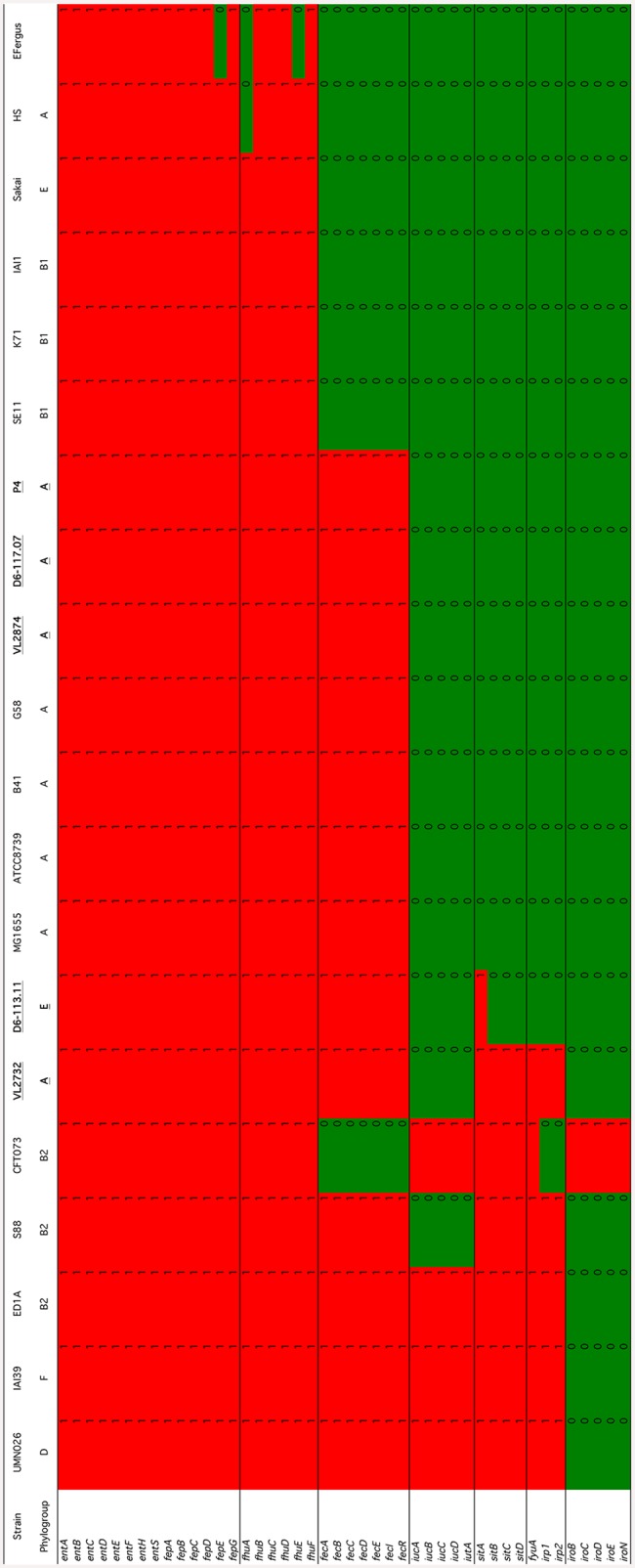
Presence/absence matrix of genes involved in iron acquisition. Presence was analyzed using tblastn (red indicates presence, green indicates absence). Mastitis isolates are in bold characters and underscored.

Another set of genes for which a role in mastitis has been suggested are those involved in iron acquisition. Indeed, iron is essential for the growth of *E*. *coli* and its transport into the bacteria requires the contribution of high affinity transport systems relying on different iron sequestering molecules called siderophore [58, 59]. At least seven iron acquisition systems have been described in the *E*. *coli* species [59]. We have thus analyzed the presence/absence of the different genes that encodes the components of these systems. Fig 6 clearly shows two categories of iron acquisition systems: the enterobactin system along with genes involved in hydroxamate uptake are present in nearly all strains tested and can thus be considered as core iron transport systems. On the contrary, the *sitABCD*, aerobactin, salmochelin, yersiniabactin and ferric citrate systems are only present in a subset of strains. When mastitis isolates are considered, except for strain VL2732, they only carry the two core systems and the ferric citrate genes.

Among the strains that carry the least number of iron acquisition genes is the K71 non pathogenic isolate as well as the commensal strains SE11, IAI1 and HS.

### Genomic incongruences

**Fig 7:**
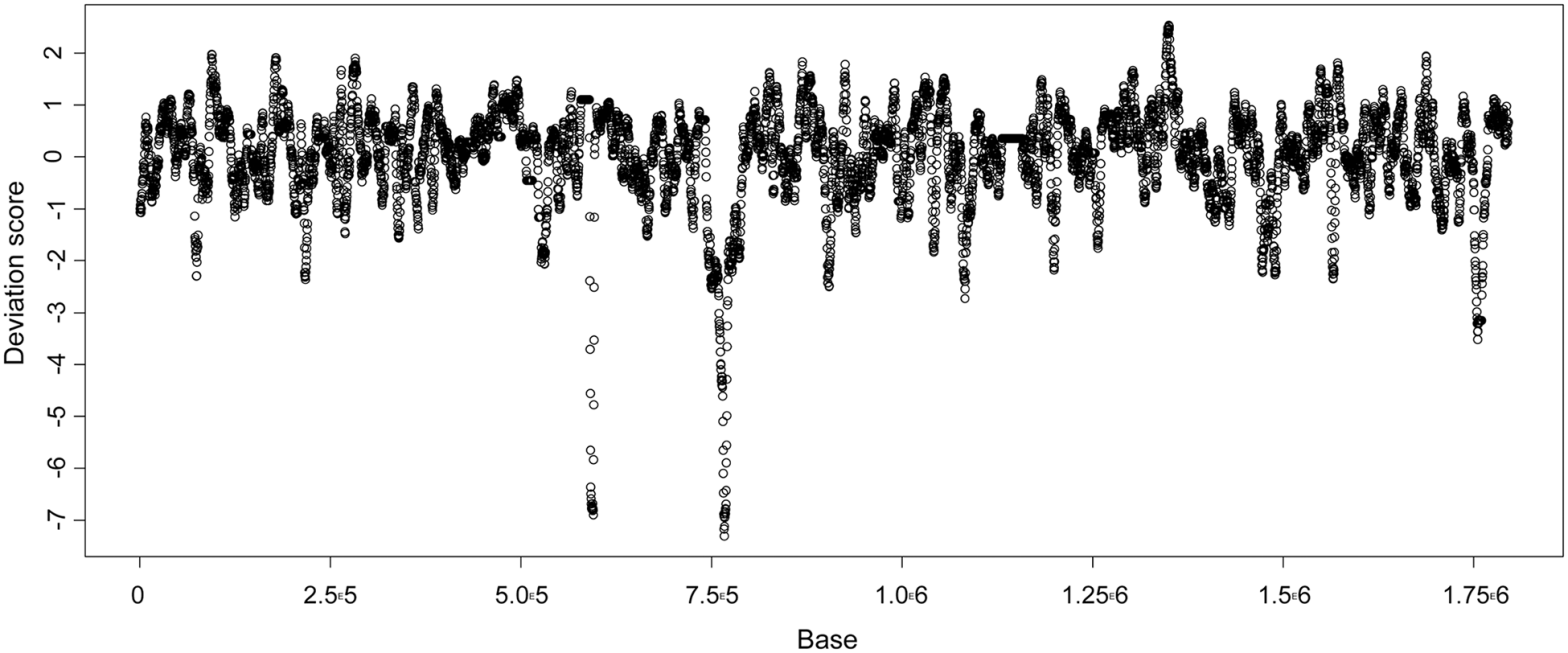
Detection of incongruence regions among the 19 genomes analyzed. The concatenated sequence of the 1976 shared genes for each of the 19 strains were compared using TreePuzzle 5.2 under HKY model for any 5kb sliding window. The sizes of incongruent regions were estimated relative to the positions of flanking genes on the *E*. *coli* EDL933 genome sequence.

We detected a total of 13 small genomic regions showing a low deviation score (S < -2) with a size ranging between 10 and 40 kb (Fig 7). In addition we found two larger regions (respectively 120kb and 275kb) showing lower deviation scores (S < -3) and centered on the *fim* and *rfb* gene clusters, respectively involved in bacterial adhesion and the O antigen synthesis. These two regions were already described as major incongruence sources but a slightly higher number of small incongruent regions (N = 23) was observed in our study [29]. The genes included in these small regions mainly coded for membrane proteins (such as AroC, ABC transporters, *rfa* locus coding for core lipopolysaccharide), putative virulence factors (fimbrial *yad* gene cluster, gene involved in iron acquisition such *fepA* and *fhuA*, toxins, invasins, other putative factor such as *pvp*). Three regions including respectively the ECA locus (Enterobacterial Common Antigen), a genomic island including the non-virulence related *phn* cluster and a region including CAS [60], and CAS genes involved in anti-viral defense were also associated with a strong incongruence. Most of them included genes involved in genetic diversification and pathogenicity.

## Discussion

The pathovar definition relies on a combination of clinical or pathogenic observations in the host and of genetic and virulence characteristics in the pathogen. As *E*. *coli* is one the primary agents of bovine mastitis, a significant effort has been made to define a new pathovar, MPEC, that would include the strains involved in this disease. Various studies have focused on identification of a specific set of virulence factors but failed to identify virulence genes specific to or significantly present in the majority of mastitis isolates [12, 13, 23, 51, 61–64]. Given the high diversity of *E*. *coli* species, mastitis *E*. *coli* isolates could include a large diversity of genetic backgrounds and various sets of virulence factors encoding for different traits determining pathogenicity [9, 12].

Strongly supported phylogenetic analyses revealed a significant variety of genetic background among mastitis strains. Our MLST-based phylogenetic analysis showed that mastitis strains seem to be mostly related to strains belonging to phylogroups A and B1 with a few strains related to strains of two other groups (D and E), these phylogroups representing the diversity of the *E*. *coli* species [65]. These results are in line with observations made in previous phylogenetic studies indicating that mastitis isolates are mainly of phylogroup A and, to some extent, of phylogroup B1 [12, 13, 51, 61, 66]. Whole-genome comparison of strains analyzed in more details in this report approach yielded similar observations and confirmed that D6-117.07, P4, VL2732 and VL2874 were related to the A group and K71 to B1 group.

The dominance of particular phylogroups among mastitis strains should be linked with studies emphasizing a relationship between phylogroup and phenotype (including pathogenicity). Commensal intestinal strains are primarily found associated with the phylogroup A [50, 67]. Among pathogenic strains, extraintestinal pathogenic *E*. *coli* (ExPEC) are usually associated with the phylogroup B2, and to a lesser extent D, whereas intestinal and less pathogenic *E*. *coli* strains predominantly derive from phylogroups A, B1, D and E [6, 67].

Most interestingly, genetic backgrounds tended to be associated with different subsets of accessory genes, as observed to some extent by others [49]. Clustering of strains based on their accessory genome is indeed very similar to that obtained with the core genome and was also described by others [68]. Clearly, this indicates that properties conferred by the accessory genome are likely to be different depending on phylogroups. This may therefore suggests that different strategies could be used by mastitis isolates of different phylogroups to trigger mastitis. One could also suggest that genetic exchanges are more frequent between strains of similar phylogroups, probably because of common lifestyle and sharing of ecological niches.

The predominance of phylogroup A strains among mastitis *E*. *coli* lends support to the hypothesis that the mastitis strain group mainly includes “typically commensal” strains of intestinal or fecal origin [12]. This hypothesis is also supported by the lack of shared virulence genes as found in our analyses of the presence of a subset of 302 recognized *E*. *coli* virulence genes.

More surprisingly, the present study reveals a relationship between phylogroup E strains and several mastitis strains. This result was confirmed for D6-113.11 using a whole-genome comparison approach. Because many phylogenetic studies are based on the initial rapid determination scheme purposed by Clermont et al. (2000), which only allow to distinguish among the four main phylogenetic groups (A, B1, B2, and D), the number of phylogroup E group strains may have been underestimated within mastitis *E*. *coli*. The E phylogroup has been historically defined by clustering a few unclassified strains [48], the most known belonging to the O157:H7 group involved in several deadly outbreaks [69]. The presence of several phylogroup E strains within the mastitis *E*. *coli* group raises questions on their virulence mechanism. The hypotheses based on a “commensal” origin pertain to D6-117.07, P4, VL2732 and VL2874, which very likely belong to the mainly commensal A. By contrast, highly virulent O157:H7 strains are known to possess specific virulence factors, including toxins, molecules responsible of the formation of secretion systems, and molecules involved in attaching/effacing lesions [70, 71]. Nevertheless, a specific search for candidate virulence genes showed that strain D6-113.11 was clearly different from the O157:H7 Sakai strain included in our comparison.

Indeed, screening for a rather extensive set of virulence genes found in the *E*. *coli* species only pointed out to a few occasions were such genes were found in mastitis isolates. Yet, no clear pattern of virulence genes could be observed. In particular, mastitis strains, apart from strain VL2874, rarely carried a significant number of genes encoding type 6 secretion systems. Similarly, genes encoding long polar fimbriae were only found in strain D6-113.11. The poor content in virulence genes of mastitis strains is best observed with the hierarchical clustering we performed: mastitis isolates are clustered with the non pathogenic *E*. *coli* K-12 MG1655 strain which clearly shows that they are different from strains belonging to the well-described IPEC and ExPEC *E*. *coli* pathovars. Yet, this does not mean that they do not carry genes that could contribute to mastitis. Although typically considered a non-pathogenic strain, the genome of K-12 MG1655 does however include a number of putative virulence genes. In fact, an MG1655 derivative carrying specific mutations in a histone-like protein, while remaining non pathogenic, showed increased invasiveness toward eukaryotic cells [72]. Thus, the similarity of mastitis strains to K-12 MG1655 in terms of gene content, which is in agreement with previous studies [14], does not exclude the possibility that mastitis strains carry genes that could promote pathogenicity in the mammary gland.

More detailed analyses of genes contributing to key properties of *E*. *coli* strains, namely LPS synthesis and iron acquisition, also did not reveal features specific for mastitis isolates. LPS is a major component of the outer membrane of *E*. *coli* and, when injected into the udder, is able to trigger an innate response that is somehow similar to the one observed during *E*. *coli* mastitis [17]. It is composed of three regions: lipid A, the core oligosaccharide and the O- antigen. Recognition of LPS by the innate immune system relies on the interaction between the host TLR4 receptor and the lipid A moiety of LPS. Interestingly, several lipid A modification enzymes have been described and, if present, could modulate the innate immune response of cows [57]. Yet, our analysis indicates that such lipid A modification enzymes are not present in mastitis isolates. In addition, strains can be classified according to the structure of their core oligosaccharide, with five different structures. Mastitis isolates analyzed in this report are predicted to have K-12, R2 or R4 core types: we cannot therefore conclude that mastitis have a preferential core oligosaccharide subtype. As to the O-antigen, previous results have already shown no association of a particular serotype with mastitis isolates [62, 73, 74].

Another property that could contribute to the growth and adaptation of *E*. *coli* in milk is the acquisition of iron. Because of its poor solubility, transport of iron requires the use of high affinity iron transport systems. Seven such systems have been described in the *E*. *coli* species. Based on our results, the two core iron transport systems (enterobactin and hydroxamate systems) along with the ferric citrate transport system are present in all mastitis isolates. Only strain VL2732 is equipped with two additional systems: the *sitABCD* operon and the yersiniabactin system, as noted previously by Blum et al. [14].

The ferric citrate system is present in all mastitis isolates, while it is absent from some other strains such as the K71 strain. Our results extend those of Blum *et al*. by showing that the entire *fec* operon, not only *fecA*, is absent in strain K71 and present in mastitis isolates. Interestingly, the K71 strain is not a mastitis isolate and was unable to trigger mastitis in murine [14] and bovine models (S. Blum, Personnal communication). One hypothesis is therefore that the ferric citrate system is required for growth in milk and that, given the high concentration of citrate in milk [75, 76], there is no need for other accessory iron acquisition genes.

In addition to the candidate gene-based approach, we attempted a bottom-up search of candidate virulence factors. A careful comparison to an *E*. *coli* pangenomic dataset allowed us to identify 36 putative novel ORFs only detected in single mastitis strains. This may be compared to the number of specific genes found within different groups of pathogenic *E*. *coli* (respectively 16 for ExPEC pathotypes strains; 28 for invasive diarrhea strain group, [29]). The functional annotation process showed that several of the genes belong to large families whose representatives are commonly involved in mechanisms of *E*. *coli* pathogenicity. These included an AraC/XylS-like regulator, belonging to a gene superfamily including several virulence factors of enteric *E*. *coli* [77, 78]; a MarR-like regulator whose superfamily is frequently involved in antimicrobial resistance regulation against agents such as tetracycline and chloramphenicol [79]; a putative adhesion protein and finally a gene coding for an O-antigen conversion protein. The remaining proteins were annotated as putative transporters, transposase genes associated with mobile elements and enzymes which implies highly diverse functional roles. Further functional studies, including *in vivo* testing and larger samples of mastitis strains will be necessary to assess the role of these genes in mastitis *E*. *coli* virulence.

In a similar fashion a genome comparison was performed to identify specific gene clusters. Although several small clusters were apparently associated with mastitis strains this likely arose from the initial choice of strains used for comparison. A broader BLAST search indeed allowed us to find close homologous genes in previously published *E*. *coli* datasets. The function of the genes included in the remaining clusters were largely unknown (23/29 genes). Among the 6 remaining genes 3 of them were likely mobile elements associated genes with unknown function; the 3 other genes respectively coding for a transcriptional regulator belonging to the AraC family, an oxygen-sensing protein (DosP) and a ClpK-like protein that may be involved in heat resistance [80]. Several phenotypic traits are expected to be associated with mammary infection strains. This include neutrophil killing evasion [9, 66] that may imply several avoidance strategies based on oxygen sensing [81]. The precise role of the genes included in these clusters is nevertheless worth further investigations. It must be noted however that mastitis specific gene clusters identified here were those not present in strains from other pathovars. However, the pathogenic potential of those strains in the mammary gland is actually unknown, implying that shared gene clusters could still have a potential role in mastitis pathogenesis.

A fine-scale analysis was finally undertaken in order to assess individual genes’ evolutionary history. Incongruences may be caused by different biological processes such as drift, selection, lateral gene transfer [82]. In *E*. *coli*, although most of the genome is clonally inherited, the presence of recombination hotspots is expected to cause local incongruences [29]. The signature of such evolutionary events was however not detected after a phylogenetic comparison integrating mastitis strains. A higher recombination was however detected around loci already known to be involved in virulence and diversification [29].

The present paper sheds a new light on the diversity that may be observed within the bovine mastitis *E*. *coli*. Taken as whole, our analyses suggest that mastitis isolates are rather characterized by the lack of genes identified as virulence determinants in other *E*. *coli* pathovars [9, 66]. Further studies should entail the whole diversity of this group with the aim of identifying mastitis-related specific gene content. This should imply full sequencing approaches, allowing a better assessment of genomic polymorphism, and larger genome samples reflecting the diversity of clinical cases as well as experimental evidence on the relative capacity of strains to cause (or not) mastitis. For now the present study is an attempt of a whole genome comparison within this *E*. *coli* group of major veterinary interest, which has been purposed to represent a distinct pathovar (MPEC). Several studies have already tackled the limits of the pathovar concept using comparative genomics approach, most of them revealing a low specific genes diversity. In line with these results, mastitis *E*. *coli* show heterogeneous genomic background, and only a few genes identified in the present study may be considered as putative virulence factors. Their precise functional role should be investigated taking into account the known mechanisms of bovine mastitis infection.

## Supporting Information

S1 Table List of *E*. *coli* virulence genes. (XLSX)

S2 Table List of mastitis related gene clusters. (DOCX)

S3 Table List of putative genes specific for a single mastitis strain. (DOCX)
