# Supplementary material for: Genomic Comparative Study of Bovine Mastitis Escherichia coli
Source: PLoS One. 2016 Jan 25;11(1):e0147954. doi: 10.1371/journal.pone.0147954 (PMC4725725; doi:10.1371/journal.pone.0147954)
Supplement: S2 Table — (DOCX) [file pone.0147954.s002.docx]

**S2 Table. List of mastitis related gene clusters**

| Locus tags (strain) ^a^ | Products | Putative protein sizes |
| --- | --- | --- |
| **CDP68555 (D6-113.11) ; EcVL2874#687 (VL2874)** | uncharacterized protein (VL2874) ; putative uncharacterized protein (D6-113.11) | 309 (VL2874) ; 309 (D6-113.11) |
| **CDP74551 (D6-117.07) ; EcVL2874#3228 (VL2874)** | uncharacterized protein (VL2874) ; putative uncharacterized protein (D6-117.07) | 359 (VL2874) ; 355 (D6-117.07) |
| EIF16388 (P4) ; CDP64129 (D6-113.11) | conjugal transfer entry exclusion protein TraS (P4) ; TraS (D6-113.11) | 173 (P4) ; 173 (D6-113.11) |
| EIF16397 (P4) ; CDP64145 (D6-113.11) | hypothetical protein (P4) ; secreted protein (D6-113.11) | 103 (P4) ; 103 (D6-113.11) |
| EIF15733 (P4) ; CDP64427 (D6-113.11) | hypothetical protein (P4) ; uncharacterized membrane protein YuaF (D6-113.11) | 200 (P4) ; 200 (D6-113.11) |
| EIF15745 (P4) ; CDP64503 (D6-113.11) | hypothetical protein (P4) ; uncharacterized membrane protein YuaP (D6-113.11) | 86 (P4) ; 86 (D6-113.11) |
| EIF18356 (P4) ; CDP67714 (D6-113.11) | hypothetical protein (P4) ; uncharacterized protein (D6-113.11) | 623 (P4) ; 623 (D6-113.11) |
| EIF18357 (P4) ; CDP67715 (D6-113.11) | hypothetical protein (P4) ; uncharacterized protein (D6-113.11) | 140 (P4) ; 140 (D6-113.11) |
| EIF18059 (P4) ; CDP68106 (D6-113.11) | hypothetical protein (P4) ; uncharacterized protein (D6-113.11) | 198 (P4) ; 198 (D6-113.11) |
| EIF18023 (P4) ; CDP68484 (D6-113.11) | hypothetical protein (P4) ; putative uncharacterized protein rth37 (D6-113.11) | 119 (P4) ; 119 (D6-113.11) |
| EIF15728 (P4) ; CDP70071 (D6-117.07) | putative transposon resolvase (P4) ; R46 site-specific recombinase (D6-117.07) | 183 (P4) ; 190 (D6-117.07) |
| EIF20033 (P4) ; CDP70963 (D6-117.07) | hypothetical protein (P4) ; putative uncharacterized protein (D6-117.07) | 127 (P4) ; 127 (D6-117.07) |
| CDP68491 (D6-113.11) ; EcVL2874#166 (VL2874) | transcriptional regulator, AraC family (VL2874) ; transcriptional regulator, AraC family (D6-113.11) | 247 (VL2874) ; 247 (D6-113.11) |
| CDP68554 (D6-113.11) ; EcVL2874#588 (VL2874) | uncharacterized protein (VL2874) ; uncharacterized protein (D6-113.11) | 445 (VL2874) ; 445 (D6-113.11) |
| CDP71504 (D6-117.07) ; EcVL2732#967 (VL2732) | oxygen sensor protein DosP (VL2732) ; putative sensor kinase (D6-117.07) | 199 (VL2732) ; 44 (D6-117.07) |
| CDP70062 (D6-117.07) ; EcVL2732#82 (VL2732) | ClpK (VL2732) ; ATP-dependent Clp protease ATP-binding subunit (D6-117.07) | 912 (VL2732) ; 949 (D6-117.07) |
| CDP70174 (D6-117.07) ; EcVL2732#315 (VL2732) | uncharacterized protein (VL2732) ; uncharacterized protein (D6-117.07) | 93 (VL2732) ; 93 (D6-117.07) |
| CDP74554 (D6-117.07) ; EcVL2874#3216 (VL2874) | MrfJ protein (VL2874) ; MrfJ protein (D6-117.07) | 125 (VL2874) ; 125 (D6-117.07) |
| CDP74555 (D6-117.07) ; EcVL2874#3149 (VL2874) | uncharacterized protein (VL2874) ; uncharacterized protein (D6-117.07) | 194 (VL2874) ; 194 (D6-117.07) |
| CDP74556 (D6-117.07) ; EcVL2874#3113 (VL2874) | uncharacterized protein (VL2874) ; putative uncharacterized protein (D6-117.07) | 239 (VL2874) ; 183 (D6-117.07) |
| CDP74561 (D6-117.07) ; EcVL2874#3027 (VL2874) | uncharacterized protein (VL2874) ; site-specific recombinase, phage integrase family (D6-117.07) | 420 (VL2874) ; 420 (D6-117.07) |
| EcVL2732#1369 (VL2732) ; EcVL2874#1754 (VL2874) | uncharacterized protein (VL2732) ; uncharacterized protein (VL2874) | 301 (VL2732) ; 301 (VL2874) |
| EcVL2732#1385 (VL2732) ; EcVL2874#1773 (VL2874) | uncharacterized protein (VL2732) ; uncharacterized protein (VL2874) | 609 (VL2732) ; 701 (VL2874) |
| EcVL2732#1487 (VL2732) ; EcVL2874#1874 (VL2874) | uncharacterized protein (VL2732) ; uncharacterized protein (VL2874) | 90 (VL2732) ; 90 (VL2874) |
| EcVL2732#1532 (VL2732) ; EcVL2874#1935 (VL2874) | uncharacterized protein (VL2732) ; uncharacterized protein (VL2874) | 77 (VL2732) ; 146 (VL2874) |
| EcVL2732#1545 (VL2732) ; EcVL2874#1948 (VL2874) | uncharacterized protein (VL2732) ; uncharacterized protein (VL2874) | 317 (VL2732) ; 317 (VL2874) |
| EcVL2732#4270 (VL2732) ; EcVL2874#716 (VL2874) | uncharacterized protein (VL2732) ; uncharacterized protein (VL2874) | 83 (VL2732) ; 83 (VL2874) |

^(a)^ Genes listed in bold do not have close homologous genes reported after BLASTP analysis.
