# Supplementary material for: Genomic Comparative Study of Bovine Mastitis Escherichia coli
Source: PLoS One. 2016 Jan 25;11(1):e0147954. doi: 10.1371/journal.pone.0147954 (PMC4725725; doi:10.1371/journal.pone.0147954)
Supplement: S3 Table — (DOCX) [file pone.0147954.s003.docx]

**S3 Table. List of putative genes specific for a single mastitis strain.**

| Strain | Locus tag | Product | Putative protein size | Most similar sequence | % identity | % positives | %  gaps | hit length |
| --- | --- | --- | --- | --- | --- | --- | --- | --- |
| D6-113.11 | PGA_04378 | Transposase | 48 | gb\|EIG68409.1\| hypothetical protein ESBG_04764 [Escherichia sp. 4_1_40B] | 100% | 100% | 0% | 38 |
| VL2732 | VL2732_02518 | Protein of unknown function | 208 | ref\|WP_031945906.1\| hypothetical protein [Acinetobacter baumannii] | 48% | 61% | 0% | 208 |
|  | VL2732_02574 | Putative uncharacterized protein | 70 | gb\|EFJ95672.1\| hypothetical protein HMPREF9540_04309 partial [Escherichia coli MS 115-1] | 90% | 92% | 0% | 62 |
|  | VL2732_02533 | Protein of unknown function | 96 | ref\|WP_044695825.1\| esterase partial [Escherichia coli] | 100% | 100% | 0% | 72 |
|  | VL2732_02517 | Uncharacterized protein | 92 | ref\|WP_001414966.1\| hypothetical protein partial [Escherichia coli] | 100% | 100% | 0% | 67 |
|  | VL2732_02515 | Putative uncharacterized protein ORF43 | 156 | ref\|WP_032490897.1\| hypothetical protein [Yersinia enterocolitica] | 99% | 99% | 0% | 101 |
|  | VL2732_02528 | Protein of unknown function | 72 | ref\|WP_032249006.1\| transposase partial [Escherichia coli] | 100% | 100% | 0% | 44 |
|  | VL2732_02536 | KAP family P-loop domain protein | 395 | gb\|EII74311.1\| KAP family P-loop domain protein [Escherichia coli 3.2303] | 100% | 100% | 0% | 237 |
|  | VL2732_02561 | Protein of unknown function | 165 | pdb\|1ECM\|A Chain A Atomic Structure Of The Buried Catalytic Pocket Of Escherichia coli Chorismate Mutase | 99% | 100% | 0% | 99 |
| VL2874 | VL2874_04000 | Uncharacterized protein | 191 | ref\|WP_042973169.1\| hypothetical protein partial [Escherichia coli] | 38% | 56% | 9% | 171 |
|  | VL2874_01354 | AraC-family trancriptional regulator | 283 | ref\|WP_024252306.1\| hypothetical protein partial [Escherichia coli] | 98% | 99% | 0% | 253 |
|  | VL2874_01356 | Phosphate transporter family protein | 192 | gb\|EFZ39332.1\| short-chain-fatty-acid--CoA ligase domain protein [Escherichia coli EPECa14] | 99% | 100% | 0% | 162 |
|  | VL2874_02752 | Short-chain-fatty-acid--CoA ligase domain protein | 188 | ref\|WP_044707763.1\| ATPase P [Escherichia coli] | 99% | 99% | 0% | 146 |
|  | VL2874_00647 | Putative uncharacterized protein | 311 | ref\|WP_032221751.1\| spermidine/putrescine ABC transporter permease [Escherichia coli] | 100% | 100% | 0% | 237 |
|  | VL2874_03007 | Protein of unknown function | 207 | ref\|WP_024184952.1\| transposase [Escherichia coli] | 100% | 100% | 0% | 149 |
|  | VL2874_02715 | Putative transposase | 76 | ref\|WP_045173901.1\| hypothetical protein partial [Escherichia coli] | 91% | 91% | 0% | 54 |
|  | VL2874_01260 | EAL domain protein | 213 | ref\|WP_045177759.1\| membrane protein partial [Escherichia coli] | 99% | 99% | 0% | 144 |
|  | VL2874_00797 | Protein of unknown function | 76 | gb\|ENG07935.1\| marR family protein [Escherichia coli P0305260.4] | 98% | 100% | 0% | 45 |
|  | VL2874_03409 | MarR family protein | 129 | gb\|EFM54577.1\| putative transposase [Escherichia coli NC101] | 99% | 99% | 0% | 73 |
|  | VL2874_01834 | Protein of unknown function | 167 | ref\|WP_042973169.1\| hypothetical protein partial [Escherichia coli] | 79% | 86% | 2% | 81 |
|  | VL2874_03872 | Transposase for insertion sequence element IS21 domain protein | 64 | >gb\|EZE10423.1\| transposase [Escherichia coli O121:H7 str. 2009C-3299] | 100% | 100% | 0% | 56 |
| P4 | UWO_16720 | hypothetical protein | 304 | gb\|EQZ08958.1\| hypothetical protein G973_04822, partial [Escherichia coli UMEA | 95% | 98% | 0% | 273 |
|  | UWO_18435 | antirestriction protein | 117 | gb\|EYD78757.1\| intergenic-region protein [Escherichia coli 1-176-05_S1_C1] | 70% | 77% | 2% | 94 |
|  | UWO_23822 | autotransporter/adhesin | 71 | gb\|KHG99492.1\| hypothetical protein PU69_28335, partial [Escherichia coli] | 98% | 98% | 0% | 57 |
|  | UWO_04266 | hypothetical protein | 93 | ref\|WP_000705363.1\| hypothetical protein [Escherichia coli] | 96% | 97% | 0% | 73 |
|  | UWO_18655 | hypothetical protein | 352 | ref\|WP_032216784.1\| hypothetical protein [Escherichia coli] | 98% | 98% | 0% | 272 |
|  | UWO_10033 | hypothetical protein | 836 | ref\|WP_033545495.1\| hypothetical protein, partial [Escherichia coli] | 100% | 100% | 0% | 590 |
|  | UWO_06115 | integrase | 351 | ref\|WP_050545092.1\| hypothetical protein [Escherichia coli] | 99% | 100% | 0% | 212 |
|  | UWO_00950 | hypothetical protein | 86 | ref\|WP_000610536.1\| hypothetical protein [Escherichia coli] | 63% | 83% | 0% | 24 |
|  | UWO_10028 | hypothetical protein | 854 | ref\|WP_033545339.1\| hypothetical protein [Escherichia coli] | 100% | 100% | 0% | 186 |
|  | UWO_09953 | hypothetical protein | 1300 | ref\|WP_032191400.1\| DNA-binding protein [Escherichia coli] | 30% | 52% | 7% | 1283 |
|  | UWO_09948 | ADP-ribosylation/Crystallin J1 | 561 | emb\|CDI19276.1\| putative ADP-ribosylation/Crystallin J1 [Klebsiella pneumoniae subsp. pneumoniae T69] | 85% | 93% | 0% | 561 |
|  | UWO_09943 | hypothetical protein | 221 | gb\|AEJ97531.1\| hypothetical protein KPN2242_08065 [Klebsiella pneumoniae KCTC] | 88% | 93% | 0% | 221 |
|  | UWO_00920 | O-antigen conversion protein | 283 | ref\|WP_032986461.1\| hypothetical protein [Cronobacter malonaticus] | 30% | 50% | 2% | 280 |
|  | UWO_22245 | cell filamentation protein | 34 | ref\|WP_032718164.1\| cell division protein Fic [Escherichia coli] | 88% | 94% | 0% | 34 |
|  | UWO_00915 | prophage bactoprenol glucosyl transferase | 60 | ref\|WP_001587941.1\| bactoprenol glucosyl transferase [Escherichia coli] | 88% | 95% | 0% | 59 |
